# NDRG4 hypermethylation is a potential biomarker for diagnosis and prognosis of gastric cancer in Chinese population

**DOI:** 10.18632/oncotarget.14099

**Published:** 2016-12-22

**Authors:** Xiaoying Chen, Yong Yang, Jing Liu, Bin Li, Yan Xu, Cong Li, Qi Xu, Guili Liu, Yingmin Chen, Jieer Ying, Shiwei Duan

**Affiliations:** ^1^ Medical Genetics Center, School of Medicine, Ningbo University, Ningbo, Zhejiang 315211, China; ^2^ Department of Medical Oncology, Zhejiang Cancer Hospital, Hangzhou, Zhejiang 310022, China

**Keywords:** gastric cancer, N-Myc downstream regulated gene 4, DNA methylation, prognosis, diagnosis

## Abstract

In order to assess whether N-Myc downstream regulated gene 4 (*NDRG4*) methylation was associated with the diagnosis and prognosis of gastric cancer, we measured the methylation of *NDRG4* promoter and gene body regions among 110 gastric cancer patients using quantitative methods (MethyLight and pyrosequencing). Both *NDRG4* promoter and gene body methylation levels were increased in tumor tissues than paired adjacent normal tissues (*P* < 0.001). *NDRG4* gene body methylation was found to be significantly associated with age and tumor differentiation. *NDRG4* promoter hypermethylation was proved to be a predictor of poor overall survival. However, opposite result was observed among The Cancer Genome Atlas (TCGA) cohort. The findings from gastric cell lines and public databases have suggested that *NDRG4* methylation level was inversely associated with *NDRG4* transcription level. Subsequent luciferase reporter gene assay showed that promoter CpG island but not gene body CpG island was able to upregulate gene expression. Collectively, *NDRG4* promoter hypermethylation contributed to the risk of gastric cancer and predicted a poor prognosis in Chinese gastric cancer patients. Moreover, the combined methylation levels of *NDRG4* promoter and gene body served as diagnostic biomarkers in gastric cancer.

## INTRODUCTION

Gastric cancer is the third leading cause of cancer-related mortality and the fourth most prevalent cancer in the world [1]. In China, gastric cancer is the second commonly diagnosed cancer, accounting for 679,100 novel cases and 498,000 cancer-associated mortalities from 2009 to 2011 [2]. The incidence of gastric cancer has a male : female ratio of approximately 2:1 [2]. And the majority (about 90%) of gastric cancer belongs to the pathological subtype of adenocarcinoma [3]. Surgical resection remains the primary treatment for gastric cancer; however, the five-year survival rate for late stage gastric cancer rarely exceeds 5% [4, 5]. Tumor node metastasis (TNM) staging classification is the most common tool in the prediction of gastric cancer prognosis. However, the latest edition of the TNM classification couldn't satisfy the exact diagnosis due to the heterogeneous clinical behavior of gastric cancer [6, 7]. Since peritoneal dissemination and local/distal metastases often occur in the late stages of gastric cancer, early diagnosis is beneficial and critical for the prevention of gastric cancer [8, 9].

Epigenetic abnormalities are considered as a significant event in the progression of cancers, such as colorectal cancer [10], lung cancer [11], esophageal cancer [12], as well as gastric cancer [13]. DNA methylation is an important epigenetic modification, and DNA methylation of cytosine-phosphate-guanine island (CGI) leads to the inappropriate silencing of tumor suppressor genes in the cancer initiation, progress, invasion and metastasis [14]. Aberrant methylation of tumor suppressor genes in gastric tissues and blood samples has been proposed as diagnostic and prognostic markers for gastric cancer [2, 13, 15, 16].

N-Myc downstream regulated gene 4 (*NDRG4*) is located on chromosome 16q21-22.1 and contains 17 exons and 16 introns. It encodes a member of the N-myc downregulated gene family, involved in modulating cell proliferation, invasion, migration and angiogenesis in human cancers [17–20]. *NDRG4* acts as a candidate tumor suppressor gene whose expression is frequently repressed by its promoter methylation in colorectal cancer [21]. Previous study has showed that the poor outcome of patients with glioblastoma was associated with *NDRG2* methylation and reduced expression [22]. *NDRG2* and *NDRG4* belong to one subfamily based on sequence homology [23]; however, the role of *NDRG4* methylation in gastric cancer is largely unknown.

In current study, we extract available The Cancer Genome Atlas (TCGA) data for discovering potentially risk sites and assess the *NDRG4* methylation level in gastric cancer patients to determine whether *NDRG4* methylation is associated with the gastric cancer risk. Additionally, we explore the prognostic value of *NDRG4* methylation in gastric cancer patients and integrate TCGA clinical data for validating the results that we found in Chinese population.

## RESULTS

### The detection of *NDRG4* hypermethylation in gastric cancer patients

**Figure 1:**
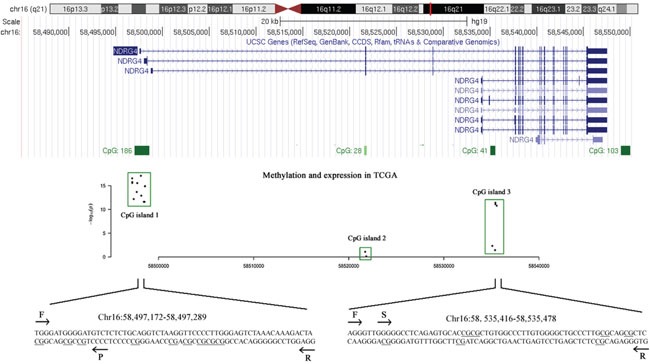
The tested CpG sites in NDRG4 promoter and gene body regions A scatter plot describes the correlation between DNA methylation profiles (Illumina Human Methylation 450K) and gene expression profiles (IlluminaHiSeq_RNA-SeqV2) obtained from TCGA datasets. The x-axis represents the genomic coordinates of NDRG4 gene CpG sites, and the y-axis represents the negative logarithm of the association P-value for each CpG site. F stands for forward primer; R stands for reverse primer; S stands for sequencing primer by pyrosequencing method; P stands for probe by MethyLight method.

Preliminary data mining in TCGA showed that the potentially risk CpG sites of *NDRG4* related with gene expression were enriched in two CGIs (CpG:192 at promoter and CpG:41 at gene body region, Figure 1). In TCGA data, there are 12 available CpG probes in promoter CGI and 5 available CpG probes in gene body CGI. All these CpG sites have a reverse correlation between DNA methylation and gene expression (all r < -0.10, *P* < 0.05, data not shown). Therefore, we chose the two fragments from promoter and gene body regions respectively for subsequent methylation test.

**Figure 2:**
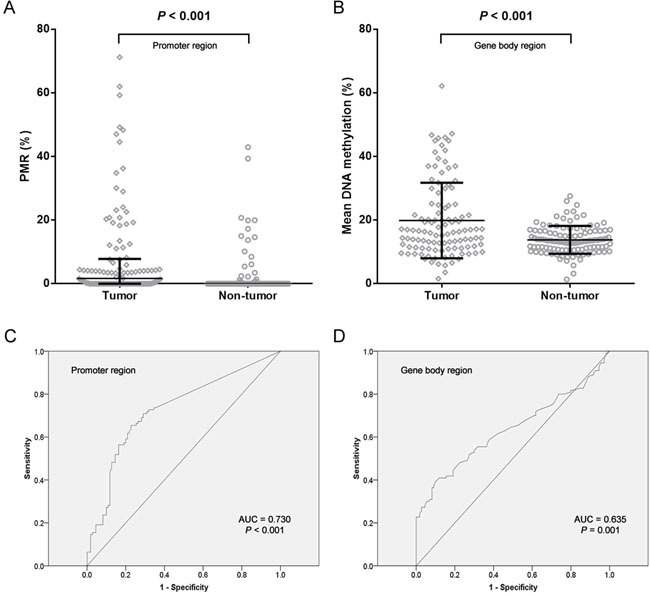
Analysis of NDRG4 gene methylation in gastric cancer patients A. Data at promoter region are presented as medians (interquartile range). B. Data at gene body region are presented as means ± standard deviation. C. ROC curve for NDRG4 promoter methylation with an AUC of 0.730. D. ROC curve for NDRG4 gene body methylation with an AUC of 0.635.

Most of previous *NDRG4* methylation studies applied methylation specific PCR method which might be biased by incompletely bisulfite-converted sequences [24]. In the present study, two quantitative methods were used to measure *NDRG4* promoter and gene body methylation levels with internal controls. We have measured the methylation of *NDRG4* CpG sites on a promoter CGI fragment (chr16:58,497,172-58,497,289) using MethyLight. We found in patients that the percent of methylated reference (PMR) of *NDRG4* promoter was significantly increased in tumor tissues than paired adjacent normal tissues (medians with interquartile range, 1.68% (0.00-7.81%) versus 0.00% (0.00-0.01%), *P* < 0.001, Figure 2A). CpG sites test on gene body CGI fragment (chr16:58,535,416-58,535,478) was using bisulfite pyrosequencing. We also observed a hypermethylated *NDRG4* gene body in tumors than matched adjacent normal tissues (mean ± standard deviation, 19.87 ± 11.88% versus 13.80 ± 4.38%, *P* < 0.001, Figure 2B). Spearman correlation showed a moderately positive relationship between promoter methylation and gene body methylation (r = 0.286, *P* = 0.002, data not shown), suggesting an overall elevated methylation level in gastric cancer. This result was compatible with the correlation between average methylation levels of HM450K CpG probes at amplified promoter (cg04190807, cg00687686 and cg04942472) and those at amplified gene body (cg11640773 and cg27102864) in TCGA data (r = 0.650, *P* < 0.001, data not shown). Meanwhile, Figure 2C and 2D showed *NDRG4* methylation was a potential diagnostic biomarker of gastric cancer (promoter methylation: 65.5% for sensitivity and 77.3% for specificity; gene body methylation: 39.1% for sensitivity and 90.0% for specificity). The combined sensitivity and combined specificity were 51.8% and 85.5%, respectively.

### Aberrant methylation with clinical phenotypes

Two detection methods (MethyLight and pyrosequencing) were applied in the promoter and gene body regions, which yielded different data distributions of DNA methylation levels. Therefore, we used different cut-off values for subsequent analyses. In Table 1, a total of 42 (38.18%) tumor tissues and 13 (11.82%) non-tumor tissues were defined as hypermethylated using the standard cutoff value of PMR is 4% [25–28] (OR = 4.609, 95% CI = 2.300-9.234, *P* < 0.001). Referring to the cut-off value of gene body methylation rate, we used the mean methylation level of tumor tissues as described previously [29]. 40 (36.36%) tumors and 9 (8.18%) normal tissues were hypermethylated (OR = 6.413, 95% CI = 2.926-14.055, *P* < 0.001).

**Table 1:** Association of *NDRG4* promoter and body methylation with clinical characteristics of gastric cancer patients

Clinical characteristics	Number	*NDRG4* promoterhypomethylation^a^	*NDRG4* promoterhypermethylation^a^	*P* value ^c^	*NDRG4* bodyhypomethylation^b^	*NDRG4* body hypermethylation^b^	*P* value ^c^
Total cases	110	68	42		70	40	
Gender				0.994			0.258
Male	76	47	29		51	25	
Female	34	21	13		19	15	
Age (years)				0.304			**< 0.001**
< 50	33	18	15		12	21	
≥ 50	77	50	27		58	19	
Tumor location				0.402			0.210
Upper	25	18	7		19	6	
Middle	25	16	9		13	12	
Lower	60	34	26		38	22	
Tumor size				0.525			0.337
< 6cm	67	43	24		45	22	
≥ 6cm	43	25	18		25	18	
Differentiation				0.243			**0.004**
High and medium	47	32	15		37	10	
Low and none	63	36	27		33	30	
Lymph node metastasis				0.240			0.307
Positive	94	56	38		58	36	
Negative	16	12	4		12	4	
TNM stage				0.058			0.517
I +II	17	14	3		12	5	
III+ IV	93	54	39		58	35	
Borrmann type				0.304 *			0.058 *
I +II	5	2	3		1	4	
III+ IV	105	66	39		69	36	
Drinking history				0.555			0.591
Yes	28	16	12		19	9	
No	82	52	30		51	31	
Smoking history				0.279			0.195
Yes	30	21	9		22	8	
No	80	47	33		48	32	
Disease recurrence				0.746			0.093
Yes	20	13	7		16	4	
No	90	55	35		54	36	

Bold value indicates statistical significance. ^a^ Cutoff value of *NDRG4* hypermethylation is set at 20%. ^b^ Cutoff value of PMR is set at 4%. ^c^ Pearson χ^2^ test, unless otherwise noted. * Fisher's exact test.

Patients were divided into younger (<=50 years) and older (>50 years) according to a 4282 patients-based retrospective study which described that early-onset gastric cancer before age of 50 was associated with family history and needed to start screening earlier [30]. Our results showed that *NDRG4* gene body were more frequently hypermethylated in the tumors of people aged younger than 50 years than those older than 50 years (63.64% versus 24.68%, *P* < 0.001, Table 1). Meanwhile, *NDRG4* gene body hypermethylation was significantly found in lower differentiation than high/medium differentiation (47.62% versus 21.28%, *P* = 0.004). However, *NDRG4* hypermethylation was not associated with other parameters, such as gender, tumor location, tumor size, lymph node metastasis, TNM stage, Borrmann type, drinking history, smoking history and disease recurrence (*P* > 0.05).

### Survival analysis

**Figure 3:**
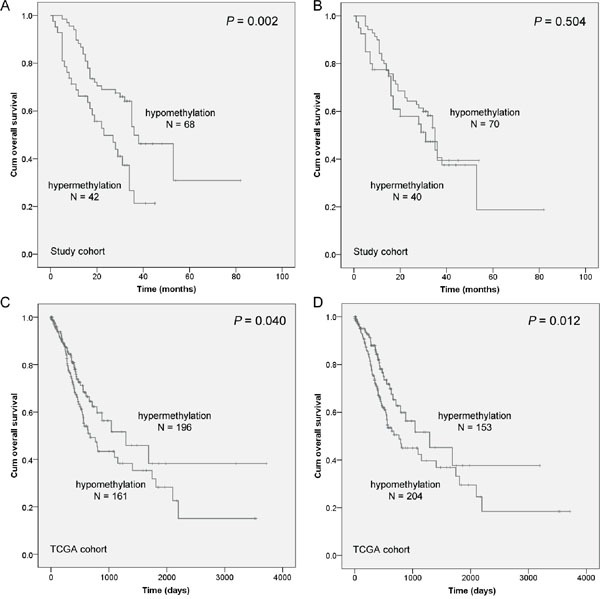
The association between NDRG4 methylation level and the prognosis of gastric cancer patients Prognostic role of A. NDRG4 promoter methylation and B. gene body methylation levels from our cohort in Kaplan-Meier curves. A cutoff value of PMR is set at 4%. A cutoff value of gene body methylation rate is set at 20%. Prognostic role of C. NDRG4 promoter methylation and D. gene body methylation levels from TCGA cohort in Kaplan-Meier curves. A cutoff values is set at the median of methylation level.

In the current study, the 5-year overall survival (OS) rate of 110 gastric cancer patients was 25.10%. As shown in Table 2, Kaplan-Meier survival analysis showed that six clinicopathological characteristics were significantly associated with OS, including age (*P* = 0.001), tumor size (*P* = 0.007), lymph node metastasis (*P* = 0.004), TNM stage (*P* = 0.001), disease recurrence (*P* = 0.009), and *NDRG4* promoter hypermethylation (*P* = 0.002, Figure 3A). These potentially important factors in univariate analyses were included in multivariate analysis. After being adjusted in a Cox proportional hazard model, age at diagnosis was a strongly independent predictor for gastric cancer prognosis (*P* = 0.007). More importantly, *NDRG4* promoter hypermethylation was shown to be associated with poor OS of gastric cancer (HR = 1.881, 95% CI = 1.107-3.218, *P* = 0.020, Table 2). However, *NDRG4* gene body methylation was not shown to be associated with gastric cancer prognosis (*P* = 0.504, Figure 3B). In TCGA cohort of 357 gastric cancer patients, Cox regression analysis revealed an inconsistent result that patients with *NDRG4* hypermethylation had a better prognosis (promoter: *P* = 0.040, Figure 3C; gene body: *P* = 0.012, Figure 3D).

**Table 2:** Independent predictors of patients’ overall survival by multivariate analysis

Clinical characteristics	Median OS (months)	χ^2^ value	Univariate*P* value	Hazard ratio (95%CI)	Multivariate*P* value *
Gender					
Male / Female	34 / 35	0.060	0.806		
Age (years)					
< 50 / ≥ 50	NA / 30	11.994	**0.001**	2.933 (1.343-6.406)	**0.007**
Tumor location					
Upper / Middle / Lower	28 / 22 / 36	5.651	0.059		
Tumor size					
<6cm / ≥ 6cm	36 / 19	7.267	**0.007**	1.464 (0.857-2.503)	0.163
Differentiation					
Low and none / High and medium	31 / 36	1.235	0.266		
Lymph node metastasis					
Negative / Positive	31 / NA	8.422	**0.004**	3.117 (0.695-13.987)	0.138
TNM stage					
I+II / III+IV	NA / 30	11.972	**0.001**	2.793 (0.598-13.049)	0.192
Borrmann type					
I+II / III+IV	NA / 34	1.231	0.267		
Drinking history					
No / Yes	35 / 34	0.020	0.887		
Smoking history					
No / Yes	34 / 35	1.486	0.223		
Disease recurrence					
No / Yes	35 / 18	6.753	**0.009**	1.388 (0.764-2.524)	0.282
*NDRG4* promoter		9.204	**0.002**	1.887 (1.107-3.218)	**0.020**
Hypomethylation / Hypermethylation	36 / 23				
NDRG4 body		0.446	0.504		
Hypomethylation / Hypermethylation	35 / 31				

Bold value indicates statistical significance. * Potentially important factors in univariate analyses (*P* < 0.05) are included in multivariate analysis. Each former charactvbfferistic is set as the reference category for hazard ratio value. OS: overall survival; NA: not available for censored data.

### The correlation between promoter methylation and gene expression

**Figure 4:**
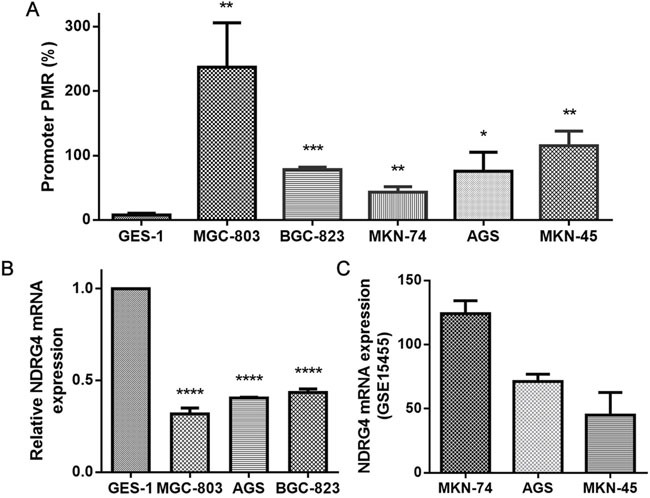
The evaluation of DNA methylation and gene expression in cell lines A. NDRG4 promoter methylation levels in GES-1, MGC-803, BGC-823, MKN-74, AGS and MKN-45 cell lines. Each reaction was performed in triplicate. * P < 0.05, ** P < 0.01, *** P < 0.001, each gastric cancer cell line versus GES-1, statistical analysis was done using independent sample t test. B. Relative NDRG4 mRNA expression in GES-1, MGC-803, BGC-823 and AGS cell lines. Each reaction was performed in triplicate. **** P < 0.0001, each gastric cancer cell line versus GES-1 (set at 1), statistical analysis was done using independent sample t test. C. The mRNA expression of MKN-74, AGS and MKN-45 was obtained from GEO database (accession number GSE15455).

In order to provide a strong evidence for validating the negative regulation of promoter methylation on gene expression, we performed two parts in cell level. Firstly, we measured *NDRG4* promoter methylation levels in five human gastric cancer cell lines (MKN-74 from well differentiated adenocarcinoma; MKN-45, MGC-803, BGC-823 and AGS, from poorly differentiated adenocarcinoma) and non cancerous gastric mucous cell (GES-1). The result showed that significantly higher *NDRG4* promoter methylation levels in gastric cell lines were all observed compared to normal cell lines (fold change = 5.578-30.607, all *P* < 0.05, Figure 4A). Meanwhile, *NDRG4* mRNA expression was detected in MGC-803, AGS, BGC-823, and GES-1 cell lines using quantitative real-time PCR (qPCR). Compared with the *NDRG4* mRNA level in GES-1 (set at 1), the relative expression levels of *NDRG4* in MGC-803, AGS and BGC-823 were all decreased (0.318 ± 0.032, 0.403 ± 0.005, 0.434 ± 0.020, respectively; Figure 4B). Secondly, with the data mining of Gene Expression Omnibus (GEO) database (accession number GSE15455), we have found a negative correlation between *NDRG4* promoter methylation and expression (Figure 4A and 4C).

**Figure 5:**
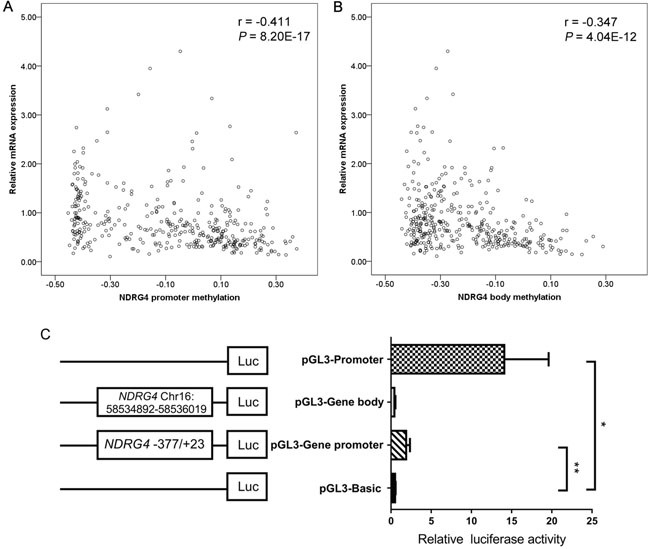
The functional role of NDRG4 in gastric cancer A. Correlation between NDRG4 promoter average methylation (cg04190807, cg00687686, cg04942472) and gene expression in 377 gastric adenocarcinoma patients from the TCGA data portal. B. NDRG4 gene body average methylation (cg11640773, cg27102864) and gene expression in 377 gastric adenocarcinoma patients from the TCGA data portal. C. Dual-luciferase reporter assay in HEK-293T cell line. The pGL3 Basic and Promoter vectors are used as negative and positive control respectively. Relative luciferase activity is performed in triplicates. Bars represent the means ± standard deviation of three independent experiments. * P < 0.05, pGL3 Promoter vector versus pGL3 Basic vector; ** P < 0.01, pGL3-NDRG4 promoter vector (-377bp to +23bp) versus pGL3 Basic vector.

Moreover, average methylation levels of CpG probes were inversely associated with gene mRNA expression in TCGA data (promoter: r = -0.411, *P* < 0.001, Figure 5A; gene body: r = -0.347, *P* < 0.001, Figure 5B). Subsequently, two dual-luciferase reporter vectors containing tested promoter and gene body fragments were identified by enzyme digestion and DNA sequencing. The result showed a significantly higher activity of *NDRG4* promoter specific region (-377bp to +23bp) but not gene body region when compared with pGL3 Basic (fold change = 3.702, *P* = 0.007, Figure 5C).

## DISCUSSION

Members of the NDRG family have been reported to be important to tumorigenesis and tumor progression in recent years [31]. The study of *NDRG4* and cancer is gaining more and more attention, although discrepant results have been observed. Schilling et al. [32] have found that NDRG4 was elevated in glioblastoma compared to human cortex tissues and NDRG4 knockdown reduces the cell viability of glioblastoma cells. However, Ding et al. [18] have held the view that NDRG4 was downregulated at both mRNA and protein levels in glioblastoma tissues compared to normal brain tissues. Chu et al. [33] reported that *NDRG4* may play its tumor suppressive role in carcinogenesis and progression through attenuation of PI3K-AKT activity in colorectal cancer. In contrast, Kotipatruni et al. [19] demonstrated that NDRG4 downregulation resulted in decreased cell proliferation, migration and invasion in aggressive meningiomas cell lines, revealing an oncogenic role in meningioma carcinogenesis. Therefore, we tend to explore the association of *NDRG4* with gastric cancer in Chinese.

Previous *NDRG4* methylation studies were mainly involved in colorectal cancer [20, 21, 34–36] and pancreatic cancer [37, 38]. Significantly increased *NDRG4* promoter methylation levels were observed in colorectal cancer tissues compared to paired non-tumor tissues [34, 35]. Meanwhile, *NDRG4* methylation might serve as an early detective biomarker in pancreatic cancer [37, 38]. In the present study, we reported for the first time that *NDRG4* promoter hypermethylation served as a predictive biomarker in gastric cancer in spite of moderate sensitivity and specificity. Currently, the gastroscopic biopsy is the gold standard for diagnosis of gastric cancer [39]. Since the approach is limited for its invasiveness and the results are always influenced by the operator's experience, strategies for gastric cancer biomarker discovery tend to be urgent. Serum carcinoembryonic antigen (CEA), cancer antigen 19-9 (CA19-9), cancer antigen 72-4 (CA72-4) and pepsinogen are conventional cancer biomarkers assessed in gastric cancer [40, 41]. Liang et al. [40] has reported that the sensitivity of CEA, CA19-9, and CA72-4 in the diagnosis of gastric cancer was 20.1–27.6% individually and 48.2% jointly in a Chinese population, which suggested a lower diagnostic ability compared with our study. Serum pepsinogen detection in a meta-analysis generated an AUC of 0.76 [42], which was compatible with the AUC of 0.73 of *NDRG4* promoter hypermethylation in gastric cancer tissues. For one thing, DNA methylation as a biomarker was more effective in the serum than that in tissues among gastric cancer patients [43]; therefore, *NDRG4* methylation might have a higher diagnostic value in serum detection. For another, these gastric cancer-associated serum markers are considered to be the most frequently present in the late stage. Since DNA methylation could be detected beginning in the early stages of gastric cancer [44], it provided an easy and quick method for gastric cancer screening. In addition, assessment of the same biomarker turned to be different diagnostic abilities in high- and low-incidence regions [45]. Therefore, *NDRG4* promoter hypermethylation could exert higher potential in those high risk areas. Specimens tested in the current study were paired and obtained from the same patient, and thus we could not make a model to test the combined ROC curve. It is a complex regulatory network underlying gastric cancer. Noteworthy, it could improve the predicted efficacy if we made a suitable multivariate model by including clinical information or other important biomarkers.

Due to the limited materials, we didn't have matched gene expression of *NDRG4* in tissues. Interestingly, we took advantage of cell lines and public databases and discovered a moderately inverse methylation-expression correlation in gastric cancer. Meanwhile, dual-luciferase reporter assay has suggested that *NDRG4* promoter specific region (-377bp to +23bp) contained a potential regulatory element. According to the study of Hapgood et al. [46], we have found that the 5’-UTR and transcription start site 200 (TSS200) regions of *NDRG4* gene contains two GC-box elements (CGCCCCCGC and GCGGGGGCG), but lacks TATA- and CCAAT-box elements. The corresponding GC box-binding proteins are Zif268 [47] and NGFI-C [48], which exert positive effects on gene transcription. Since the function of GC-rich DNA is not linked to a particular cellular process or mechanism of regulation [46, 49], further relationship between *NDRG4* methylation and other cis-regulatory elements in gastric carcinogenesis is needed to be explored. Previous study suggested that CpG sites hypermethylation in the first intron of tumor suppressor gene was significantly associated with an increased risk of colorectal cancer, and the demethylation of CpG sites could restore gene expression [50]. Although several studies have investigated the mechanism of gene body methylation on gene expression [51–53], the function of gene body methylation is largely unknown. DNA methylation on gene body was shown to positively or negatively regulate gene expression [54, 55]. Our findings showed that a much higher hypermethylation rate of *NDRG4* gene body in tumor tissues than their adjacent tissues. Therefore, we speculated that *NDRG4* overall hypermethylation at promoter and gene body CGI could contribute to the risk of gastric cancer through its regulation of gene expression.

Our results observed that *NDRG4* body methylation was shown to be associated with age and differentiation. Age is a well known risk factor in the diagnosis and prognosis of gastric cancer [30, 56]. We found that the degree of methylation difference between tumor and non-tumor tissues was negatively correlated with aging, providing a clue to elaborate the epigenetics mechanism of aberrant DNA methylation in gastric cancer during aging. *NDRG4* body hypermethylation may play an important role in gastric carcinogenesis in the younger patients. Screening for *NDRG4* body hypermethylation might have clinical significance for the evaluation of younger patients with gastric cancer. In addition, those poorly differentiated tissues were inclined to accompany the *NDRG4* body methylation changes during gastric cancer progression, suggesting the potential to distinguish different stages of differentiation.

Although no significance was found in *NDRG4* promoter methylation with clinical characteristics, *NDRG4* hypermethylation was shown as a poor prognostic biomarker in Chinese gastric patients but as an improved prognostic biomarker in TCGA data. Almost all gastric cancers are adenocarcinomas, and thus we speculated this discrepancy may be due to different ethnicities, therapies, and methylation detection methods. Positive NDRG4 staining was an independent predictor of favorable survival in a cohort including 272 Chinese colorectal cancer (CRC) patients [33]; however, we could not find the support evidence from 174 CRC patients in TCGA4U [57], a web-based platform using TCGA datasets (P > 0.05, data not shown). Moreover, our previous work has showed that different ethnic groups existed diverse effects of gene methylation [58]. Taking the prognosis studies of malignant glioma for instance, we have found that *MGMT* promoter methylation was associated with worse OS among Asians [59], whereas it was associated with longer OS among Caucasians [60]. In current study, 377 TCGA gastric patients are from Europe (100 samples), North and South America (128 samples), Asia (18 samples), and unknown region (131 samples). Therefore, ethnicity-based genetic heterogeneity should be considerable in the prognosis prediction. Further validation with larger sample size in other ethnic population is required. Different treatments always affect the outcomes of patients. Multiple agents are active in the treatment of gastric cancer, including fluoropyrimidines, platinum agents, anthracyclines, taxanes, and irinotecan [61]. It was difficult to determine whether the survival differences were caused by inherent prognostic differences or were a result of a treatment interaction, or both. In a study of a different design, Mitsuno *et al*. reported that patients with p16 methylation gained longer survival from chemotherapy, while those without methylation did not [62]. Since both TCGA data and our study cohort lacked sufficient treatment information, much further work is required to confirm our findings. Additionally, TCGA data are based on the high throughput sequencing method, and genome-scale methylation assessment alone was unlikely to inform patient outcome for oral tongue squamous cell carcinoma [63]. Therefore, it may be a shortcoming for drawing a prognostic conclusion study only relating to the HM450K platform itself.

Tumorigenesis is often associated with functional genetic variants, such as copy number alterations and loss of heterozygosity [64]. In NCBI dbSNP database, *NDRG4* has 356 indel mutations, 3059 single nucleotide polymorphisms (SNPs) and 6 multiple nucleotide polymorphisms (MNPs). Among these variations, four SNPs (rs40186, rs246192, rs11076243 and rs11862356) have been cited in previous studies [65–68]. However, the research of *NDRG4* variants in human cancers is scarce. A recent exome sequencing study [69] has identified that c.511G>C (p.Val171Leu), a novel *NDRG4* homozygous variant, is associated with the autosomal recessive form of infantile myofibromatosis, showing that *NDRG4* variations may play an important role in benign tumor. Future recent research on the interaction of genetic polymorphisms and epigenetic marks on *NDRG4* gene might be useful to elaborate the role of this gene in gastric cancer risk.

In conclusion, our findings suggested that *NDRG4* CpG island hypermethylation could be a potential biomarker for diagnosis of gastric cancer. Meanwhile, *NDRG4* promoter hypermethylation was identified as an independent prognostic factor for survival outcomes in Chinese gastric cancer patients, and it exerted a tumor suppressive role in carcinogenesis and progression through attenuation of *NDRG4* expression.

## MATERIALS AND METHODS

### Tumor samples and TCGA data source

Tumors and their paired adjacent tissues came from 110 gastric cancer patients diagnosed between January 2008 and February 2015 at the Zhejiang Province Cancer Hospital, China. All the patients were histologically verified with gastric adenocarcinoma before chemotherapy and radiotherapy. The OS was defined as the date of primary surgery to the date of death or the date of last follow-up [70]. All the specimens were freshly obtained and stored at -80°C. All of the experiments were approved by the Ethical Committees of the above mentioned hospital, and methods have been carried out in accordance with approved guidelines. All the patients had signed the informed consent forms.

For TCGA cohort, DNA methylation profiles (Illumina Human Methylation 450K, HM450K), gene expression profiles (IlluminaHiSeq_RNA-SeqV2) and clinical data generated from 377 stomach adenocarcinoma patients were available from the website of Cancer Genomics Browser of University of California Santa Cruz (UCSC) (https://genome-cancer.ucsc.edu/). Ethnic groups consist of those from Europe (100 samples), North and South Americas (128 samples), Asia (18 samples) and unknown region (131 samples).

### Cell culture

A non-cancerous gastric mucous cell (GES-1), five human gastric cancer cell lines [a well differentiated adenocarcinoma cell line (MKN-74) and four poorly differentiated adenocarcinoma (MKN-45, MGC-803, BGC-823 and AGS)], and the human embryonic kidney HEK293T cell line were obtained from the cell bank of Chinese academy of sciences (Shanghai, China). All of them were cultured at 37°C in high-glucose Dulbecco's modified Eagle's medium (DMEM, HyClone, Logan, Utah) supplemented with 10% fetal bovine serum (FBS, TransGen Biotech, Beijing, China).

### DNA isolation, bisulfite conversion, MethyLight method and pyrosequencing

Genomic DNA was extracted by the QIAamp DNA Mini Kit (Qiagen GmbH, Hilden, Germany) according to the manufacturer's instruction. DNA concentrations measure and bisulfite conversation of genomic were as previously shown [71].

MethyLight was used to measure the gene promoter methylation level in cell lines and all participants. It was performed in a total volume of 15 μL containing 7.5 μL 2 × HotTaq Master Mix (QuanRen Biotech, Shanghai, China), 0.75 μL forward and reverse primers (5 μM), 0.75 μL Taqman probe (2 μM), 3.75 μL nuclease-free water and 1.5 μL bisulphite-converted DNA. Control reference gene *ACTB* was amplified in parallel to normalize DNA input. Primer and probe sequences were listed in Table 3. MethyLight was performed on LightCycler 480 (Roche, Basel, Switzerland) under the following condition: 95°C for 10 min, followed by 45 cycles of 95 °C for 15 s and 60 °C for 45 s. EpiTect methylated control DNA (Qiagen, Hilden, Germany) was used as a positive control. Water without DNA served as a control for contamination and primer dimer in each assay. Each MethyLight reaction was performed in duplicate, and PMR values were used to quantify the methylation level of each sample [72].

**Table 3:** Oligonucleotides and amplification conditions

	Primer sequence (5'-3')	Amplicon length (bp)	Annealing temp. (°C)
Gene body-F	AGGGTTGGGGGTTTTAGA	128	55.4
Gene body-R	[Biotin]-CACCCTCTACCAAAAACTCAAAACTCAATT		
Gene body-S	GGGGTTTTAGAGTGTAT		
Promoter-F	TGGGATGGGGATGTTTTTGT	118	60
Promoter-R	CCTCCAAACCCCCTATAACC		
Promoter-P	[6FAM]-AAAACGACGCTACCGTAATCTTTA-[BHQ1]		
ACTB-F	TGGTGATGGAGGAGGTTTAGTAAGT	133	60
ACTB-R	AACCAATAAAACCTACTCCTCCCTTAA		
ACTB-P	[6FAM]-ACCACCACCCAACACACAATAACAAACACA-[BHQ1]		
*NDRG4* mRNA-F	GGCCTTCTGCATGTAGTGATCCG	385	60
*NDRG4* mRNA-R	GGTGATCTCCTGCATGTCCTCG		
Cyclophilin A-F	CTCGAATAAGTTTGACTTGTGTTT	165	60
Cyclophilin A-R	CTAGGCATGGGAGGGAACA		

F stands for forward primer; R stands for reverse primer; S stands for sequencing primer by pyrosequencing method; P stands for probe by MethyLight method.

Pyrosequencing was applied to measure the gene body methylation levels in tissues. The details of pyrosequencing procedures were as previously shown [73]. Primer sequences and PCR conditions were listed in Table 3. Average methylation levels for *NDRG4* gene body were calculated for the five CpG sites included in the assay.

### RNA extraction and QPCR method

Total RNA was isolated from MGC-803, AGS, BGC-823, and GES-1 cell lines by Qiagen RNeasy Mini kit (Qiagen GmbH, Hilden, Germany). Reverse transcription-PCR was performed with 2 μg of isolated total RNA and synthesized to cDNA in a 20 μl reaction system using reverse transcriptase (Promega, Wisconsin, USA) with oligo-dT primers according to the manufacturer's instructions. QPCR to quantify *NDRG4* mRNA level was performed by SYBR Green master mix (Roche, Basel, Switzerland). The PCR system followed the same condition of MethyLight method mentioned above. *Cyclophilin A* was used as a reference gene for normalization. Primers were listed in Table 3. Gene expression levels were calculated using the ΔCt (delta cycle threshold) method: ΔCt = mean value Ct (mRNA reference) − mean value Ct (mRNA of interest). The relative mRNA expression of *NDRG4* gene corresponded to the value 2^ΔCt^.

### Construction of recombinant plasmids

The fragment of *NDRG4* promoter (-377bp to +23bp) was chemically synthesized and the fragment of gene body was amplified with forward primer 5’-CTTACGCGTGCTAGCCCGTGGGGGAAGGCAACGCT-3’ and reverse primer 5’-CGCAGATCTCGAGCCCCCTGCCAGGTGCCAGTCTC-3’. The amplified promoter DNA fragment was digested with XhoI and NheI, and the amplified gene body fragment was digested with Mlul and BgIII (New England Biolabs, Ipswich, MA). After being purified by Cycle Pure Kit (Omega, Norcross, GA, USA), the target fragment was cloned to pGL3 Basic vector (Promega, Madison city, WI, USA) by DNA Ligation Kit (Takara, Japan).

### Plasmids transfection and luciferase reporter assay

Cells are prepared in 96-well plates and the details of plasmids transfection are as described previously [74]. After 18-72h of HEK293T cells transfection, luciferase activity is determined with the dual luciferase reporter assay system (Dual-Luciferase^®^ Reporter Assay Systems, Promega, Madison city, WI, USA). Renilla and firefly luciferase activities were measured by SpectraMax 190 (Molecular Devices, Sunnyvale, USA).

### Statistical analysis

All the statistical analyses were conducted with SPSS 18.0 software (SPSS Inc, Chicago, IL, USA) and R 3.1.2 software. Pearson χ^2^ test and Fisher's exact test were used to evaluate the association of the *NDRG4* methylation with the clinical characteristics. Wilcoxon matched pairs test and paired sample t-test were applied for the comparisons of *NDRG4* methylation between cancer tissues and their adjacent tissues. Spearman rank test was used to compute the correlation between *NDRG4* methylation and gene expression. Kaplan-Meier method and log-rank test were applied to the data of the gastric cancer patients classified into two groups according to the *NDRG4* methylation status. Cox proportional hazard model was fitted with calculating hazard ratio (HR) and the corresponding 95% confidence interval (95% CI). Receiver operating characteristic (ROC) analysis was used to compare the sensitivity and specificity by the parameters. The firefly luciferase activity was normalized to the renilla luciferase activity, and values were presented as means ± standard deviation from three single experiments. *P* < 0.05 was considered to be statistically significant.

## Abbreviations

NDRG4: N-Myc downstream regulated gene 4
TCGA: The Cancer Genome Atlas
TNM: tumor node metastasis
CGI: cytosine-phosphate-guanine island
PMR: the percent of methylated reference
OS: overall survival
qPCR: quantitative real-time PCR
GEO: Gene Expression Omnibus
SNP: single nucleotide polymorphism
MNP: multiple nucleotide polymorphism
HR: hazard ratio
CI: confidence interval
ROC: receiver operating characteristic
AUC: area under the curve
